# Current production in a microbial fuel cell using a pure culture of Cupriavidus basilensis growing in acetate or phenol as a carbon source

**DOI:** 10.1111/1751-7915.12026

**Published:** 2013-01-10

**Authors:** Hen Friman, Alex Schechter, Yulia Ioffe, Yeshayahu Nitzan, Rivka Cahan

**Affiliations:** 1 Department of Chemical Engineering and Biotechnology, Ariel University CenterAriel, 40700, Israel; 2 Department of Biological Chemistry, Ariel University CenterAriel, 40700, Israel; 3 The Mina 0026; Everard Goodman Faculty of Life Sciences, Bar-Ilan UniversityRamat-Gan, 52900, Israel

## Abstract

A microbial fuel cell (MFC) was operated with a pure culture of *Cupriavidus basilensis* bacterial cells growing in the anode compartment in a defined medium containing acetate or phenol. Operating this mediator-less MFC under a constant external resistor of 1 kΩ with acetate or phenol led to current generation of 902 and 310 mA m^−2^ respectively. In the MFC which was operated using acetate or phenol, the current density measured from the plankton bacterial cells with a fresh electrode was 125 and 109 mA m^−2^, respectively, whereas the current obtained with biofilm-covered electrodes in sterile medium was 541 and 228 mA m^−2^ respectively. After 72 h in the MFC, 86% of the initial phenol concentration was removed, while only 64% was removed after the same time in the control MFC which was held at an open circuit potential (OCP). Furthermore, SEM and confocal microscopy analyses demonstrated a developed biofilm with a live *C. basilensis* population. In conclusion, in this study we demonstrated, for the first time, use of *C. basilensis* facultative aerobe bacterial cells in a MFC using acetate or phenol as the sole carbon source which led to electricity generation.

## Introduction

Microbial fuel cells (MFCs) have long been considered an attractive mean for converting various carbohydrate wastes directly into electricity using electrogenic bacterial cells in the anode compartment. Most MFCs have been operated using anaerobic or facultative aerobic bacteria which oxidize various substrates including glucose, sewage sludge and petroleum hydrocarbon (Park and Zeikus, 2003; Min *et al*., 2005; Rabaey *et al*., 2005; Cheng *et al*., 2006; Morris and Jin, 2008). Substrates of particular interest for use in MFCs are soluble by-products of dark fermentation (Kumar *et al*., 2008) that include volatile fatty acids such as lactic, formic, butyric, propionic and succinic acids, alcohols and solvents (Lalaurette *et al*., 2009; Ren *et al*., 2009; Kiely *et al*., 2010; Liu *et al*., 2010).

Power production by MFCs varies with the specific substrate concentration, the bacterial cell species and the MFC configuration (Rabaey *et al*., 2005; Liu *et al*., 2010). Typically, MFCs which were operated with a mixture of bacterial cells produced higher specific power than MFCs operated by a monoculture in the anode compartment (Rabaey *et al*., 2005).

A two-chamber MFC in which the electrodes were connected via a 500 Ω fixed resistor was operated with a pure culture of *Geobacter sulfurreducens.* Acetate was provided as an electron donor and current production in this MFC was 16 mW m^−2^ at 65 mA m^−2^ and 0.25 V (Bond and Lovley, 2003). A MFC that was inoculated with the wild-type strain of *G. sulfurreducens*, strain DL-1, was operated for 5 months. In this MFC, an isolate strain KN400, was recovered from the biofilm of the electrode. This strain was much more effective in current production than the wild-type strain DL-1. Peak power densities obtained by KN400 and DL-1 strains were 3.9 W m^−2^ at 7.6 A m^−2^ and 0.51 V and 0.5 W m^−2^ at 1.4 A m^−2^ and 0.36 V respectively. This was obtained using ferricyanide as the oxidant in the cathode. The enhanced capacity for current production with KN400 was attributed to a greater abundance of electrically conductive microbial nanowires than in the DL-1 strain (Yi *et al*., 2009).

Phenols are among the most common industrial pollutants due to their frequent presence in the waste effluents of many industrial processes. Phenol and its derivatives are toxic to aquatic flora and fauna even at low concentrations (Agarry *et al*., 2008). Treatment of phenol effluents is therefore very important. Bioremediation methods that use microorganisms for degrading phenol contaminants into less toxic forms constitute an attractive alternative to conventional techniques (Gopaul *et al*., 1991; El-Sayed *et al*., 2003). One of the species used in the phenol bioremediation process is *Cupriavidus basilensis*, a Gram-negative flagellated aerobe, related to the β-proteobacterium (Ledrich *et al*., 2005; Fischer *et al*., 2010). Members of this family are metal-resistant and are able to degrade phenol and a wide range of aliphatic alcohols, including methanol and ethanol (Monchy *et al*., 2007).

In this research, electricity production was attained in a MFC using a pure culture of facultative aerobe *C. basilensis* bacterial cells as opposed to the conventional MFC which uses anaerobic bacteria or mixed cultures. Current production using acetate as the sole carbon source was higher than when phenol was used as the sole carbon source. However, biodegradation of phenol was achieved in the MFC. Study of MFCs using a pure culture may contribute to our understanding of the electricity production processes in this facility.

## Results and discussion

### Bacterial growth in MFC

The MFC was operated under a constant external resistance of 1 kΩ using a pure culture of *C. basilensis* bacterial cells in the anode chamber. A parallel MFC which was held at an open circuit potential (OCP) was maintained under the same conditions as the MFC. Every 100 h, 100 ml from the anode chamber was replaced by fresh minimal medium (MM) and a final concentration of 10 mM acetate or 1.06 mM phenol was added. The growth curve of *C. basilensis* grown in a defined medium containing acetate as the sole carbon source in the MFC as well as in the control MFC(OCP) was about the same. Similar results were obtained when the MFC was operated with phenol as the sole carbon source. When the MFC was operated with acetate, the cultures in the MFC and in the control MFC(OCP) reached 0.824 and 0.722 OD_600_ respectively. However, when the MFC was operated with phenol as the sole carbon source, the cultures in the MFC and in the control MFC(OCP) reached only 0.456 and 0.411 OD_600_ respectively (data not shown). It is important to indicate that in these MFC set-ups the oxygen concentration at the beginning of the experiment was 8.4 mg l^−1^, after 100 h of the MFC operation the oxygen concentration reduced to 0.8 mg l^−1^. Immediately after addition of fresh medium the oxygen concentration increased to 2.2 mg l^−1^. We assumed that the reduction of the oxygen concentration was occurred as a consequence of the bacterial metabolism.

In a MFC which was held under anaerobic conditions no bacterial growth was observed.

### Current formation in the MFC using acetate or phenol as the sole carbon and energy source

**Figure 1:**
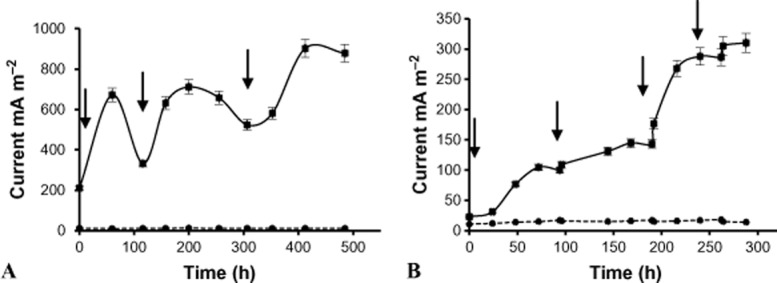
Current formation in a MFC with *C. basilensis* grown in a minimal medium with acetate (10 mM) (A); and phenol (1.06 mM) (B) as the sole carbon source (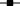) and in the abiotic controls MFC (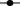). The apparent electrode surface area is 3 cm^2^. The arrows indicate acetate (A) and phenol (B) feedings.

The MFC was operated under a constant external resistance of 1 kΩ using a pure culture of *C. basilensis* bacterial cells growing in MM-Acetate (MM-A) or MM-Phenol (MM-P) in the anode chamber. A parallel abiotic MFC was maintained under the same conditions as the MFC, except for bacterial inoculation. The current generated in the MFCs and abiotic MFCs was measured continuously during the experiment. The anode surface area was only 3 cm^2^ which led to higher resistance then the applied external resistance (1 kΩ). The MFC current depends on the sum of both resistances: internal resistance (mainly, anode resistance) and external resistance (applied 1 kΩ resistance). Therefore the presented currents are normalized by the surface area of highest resistance element (the anode interfacial resistance) and not by the cathode or the external resistance. Three current peaks of 672, 717 and 902 mA m^−2^ were observed in each MFC which was operated with acetate as the sole carbon source. Each of the peaks was observed about 60 h after the acetate addition as shown in Fig. 1A. The current of 902 mA m^−2^ was maintained until the end of the experiment (500 h) (Fig. 1A). A moderate increase in the current, up to 140 mA m^−2^, was recorded from the beginning of the experiment until 188 h of operation in the MFC which was operated with phenol as the sole carbon source. However, from 188 h until the end of the experiment, the current increased sharply to 310 mA m^−2^ (Fig. 1B). Feeding phenol (1.06 mM) at 0, 94, 188 and 262 h led to an increase in the current. The current measured in the abiotic control MFCs (which were operated with acetate or phenol) without bacterial cells was negligible, indicating that electrochemical oxidation of acetate or phenol does not contribute to the current.

Columbic efficiency calculation was 54% for acetate feed MFC, based on full conversation of acetate to CO_2_. Similar mathematical treatment is not applicable to phenol-based MFC, due to the large distribution of plausible oxidation products of biodegradable phenols (van Schie and Young, 2000).

It is important to indicate that at each carbon source feeding, the MFC was introduced by fresh medium with dissolved oxygen of 8.4 mg l^−1^. We assume that in MFC using *C. basilensis* bacterial cells which require oxygen for growth the charge transfer occurs via mediator shuttle mechanism, where the dissolve electro active species produced by the bacteria is independent of the oxygen concentration in the solution. The current produced in the MFC using *C. basilensis* which was operated under low-oxygen environment (Fig. 1) is contrary to results obtained with a *Paracoccus denitrificans* strain PS-1 which was tested for its ability to function as an exoelectrogen with formic acid supplied as the electron donor. Although strain PS-1 could grew in the reactor, negligible current was produced due to oxygen leakage through the cathode (Kiely *et al*., 2010).

Current production using a monoculture is usually low compared with mixed cultures. Furthermore, MFCs that use Gram-negative bacterial cells produce a higher current than those that use Gram-positive bacteria. The current generated in batch MFCs with the Gram-negative *G. sulfurreducens*, *Pseudomonas aeruginosa* and *Shewanella oneidensis* bacterial cells was 1.0 ± 0.05, 0.9 ± 0.01 and 1.0 ± 0.15 mA m^−2^, respectively, whereas the current generated in batch MFCs with the Gram-positive *Clostridium acetobutylicum* and *Enterococcus faecium* bacterial cells was only 0.1 ± 0.03 and 0.2 ± 0.05 mA m^−2^ respectively (Read *et al*., 2010).

The MFC in which *C. basilensis* (Fig. 1) was used produced a relatively high current compared with the above-mentioned MFCs.

### Current versus voltage polarization

**Figure 2:**
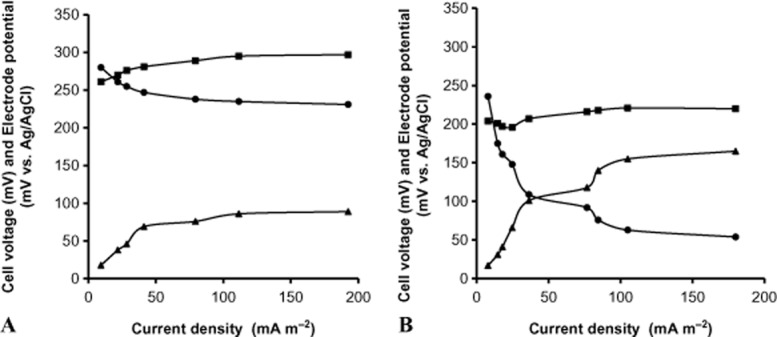
Steady-state current-potential polarization curve in the MFC using *C. basilensis*, MFC voltage (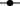), cathode potential (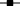) and anode potential (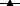) in a defined anode medium with acetate (A) and phenol (B) as the sole carbon source.

The steady-state current-potential polarization curve was measured after the cells’ voltage was stabilized in MFCs containing phenol or acetate (Fig. 2A and B). The voltage was measured between the cathode and the anode (MFC voltage) as well as between each of these electrodes and the reference electrode. As the applied external resistance decreases from 100 kΩ to 1 kΩ, the cell's current flowing between the anode and the cathode increases and the voltage across the cell diminishes. Below 1 kΩ the variations of the voltage were too small to be significant. The cathode exhibited no potential loss compared with the anode. Hence, the cathode did not contribute much to the MFC's total resistance and potential loss. In the bacterial anode on the other hand, the potential increased due to internal charge transfer resistance. The MFC losses can be attributed to this slow rate of charge transfer. However, the anode operated with phenol demonstrated higher resistances than the MFC operated with acetate (Fig. 2). The interfacial resistance (Ra) in the phenol-based cell was calculated from the slope of the anode curve: below 40 mA^2^ Ra = 3.0 Ω m^2^ and at higher currents Ra is 0.4Ω m^2^ (Fig. 2B). Higher currents and smaller anode interfacial resistance were observed in the MFC operated with acetate (*c*. Ra below 40 mA m^−2^ is 1.6 Ω m^2^ and Ra = 0.09 Ω m^2^ above this current) (Fig. 2A). These results are in line with Fig. 1, showing higher currents corresponding to faster oxidation kinetics in the acetate-based MFC. Since no mediator molecule was added to the anode chamber, the reduced kinetic of charge transfer in the anode should result from a low concentration of soluble natural mediators or direct electron transfer by the biofilm which developed on the anode. It should be noted that in typical MFCs which were described in other studies, the anode potential rests at a more negative value than the Ag/AgCl reference potential. This phenomenon is ascribed to redox coupling between NAD^+^/NADH (e.g. E^0^′ = −0.48V, E^0^′ – standard reduction potential) and served as an indication for electrogenic activity (Logan, 2008).

However, the anode potentials described in Fig. 2 are all positive with respect to the Ag/AgCl reference. We assume that the positive anode potential results from a mixed potential effect in the presence of a low oxygen concentration introduced into the MFC through the non-hermetically sealed cap and the periodically addition of fresh medium.

A shift to more positive potentials by exposure of the anode to oxygen was reported by Logan *et al*., who showed that the anode potential of mixed culture cells increased from negative potentials (−200 mV versus Ag/AgCl) to more positive potentials (∼ 0 mV) after aeration of the MFC (Oh *et al*., 2009).

The power density of the MFC was calculated from the steady-state polarization at increasing current densities. The increase in power density was correlated with the increases in current density. The maximum power density in MFC using phenol reached 10 mW m^−2^ (at 175 mA m^−2^) while in MFC using acetate it reached 44 mW m^−2^ (at 193 mA m^−2^).

Park and Zeikus (2003) studied power generation in a similar MFC configuration but with *Escherichia coli* grown in a medium containing lactate and a graphite cathode containing Fe^3+^ as oxidant. The power density calculated in this MFC was 0.30 mW m^−2^ (0.47 mA m^−2^) (Park and Zeikus, 2003). The MFC in the present study was operated using *C. basilensis* while Park and Zeikus (2003) operated the MFC using *E. coli*. Both bacterial cells *C. basilensis* and *E. coli* are facultative aerobe bacterial cells. However, the power generated by the MFC using *C. basilensis* was more than one order of magnitude higher. We attribute this behaviour to formation of a more active biofilm and a natural shuttle mediator in *C. basilensis*.

The phototrophic purple non-sulfur bacterium *Rhodopseudomonas palustris* DX-1 was one of the first used to demonstrate that pure cultures can generate currents at densities comparable to mixed communities. This isolate produced electricity at a higher power density (2720 ± 60 mW m^−2^) than mixed cultures in the same device (Xing *et al*., 2008).

However, the power density in most MFCs is much lower. A *P. denitrificans* strain PS-1 produced only 5.6 mW m^−2^, whereas the original mixed culture produced up to 10 mW m^−2^ (Kiely *et al*., 2010). The isolated *Shewanella putrefaciens* strain PS-2 was capable of producing a higher power density (17.4 mW m^−2^) and a maximum voltage of approximately 150 mV, which was higher than that of the mixed culture which reached 90 mV (Kiely *et al*., 2010).

### CV measurements in a bacterial culture

**Figure 3:**
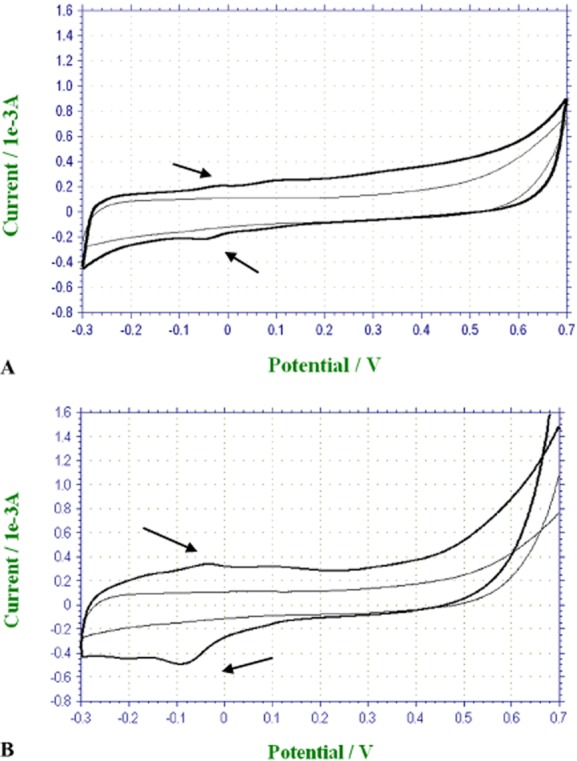
Cyclic voltammetry (graphite working electrode versus Ag/AgCl reference electrode, 10 mV s^−1^) immediately after adding the bacteria to the MFC (thin line) and at the end of the experiment MFC (using acetate) (A) and MFC (using phenol) (B).

CV measurements of the graphite electrode were performed at the beginning and end of the experiments using acetate (Fig. 3A) or phenol (Fig. 3B) as the sole carbon source. The *voltammogram* at the end of the experiment has distinct oxidation and reduction peaks compared with the same electrode at the beginning. Same redox couples were seen at the end of the experiment in the cell-free supernatant (data not shown). No obvious oxidation or reduction peaks could be detected at the initial stage of acclimation. However, a clear redox couple was detected in the MFC using acetate and MFC using phenol with E_1/2_ = −0.025 V and −0.075 V, respectively, with a peak separation of 60 mV between the oxidation and reduction, indicative of a reversible homogeneous electrochemical reaction (e.g. soluble redox). The nature of this reaction is unclear at present. However, it is postulated that there is a release or accumulation of electroactive material to the solution with time, which can readily undergo oxidation on the anode. This postulated electroactive mediator material is created as a result of the bacterial cell activity. It is important to mention that the increase in the anodic current described in Fig. 3 cannot be attributed to acetate phenol or electro-oxidation, since an insignificant current was measured on the same electrode in a sterile MM-P, indicating that phenol oxidation is negligible (data not shown).

Mediated charge transfer (MET) is suggested as a classical mechanism for current generation in MFCs. MET is ascribed to natural redox molecules or to artificial molecules that can undergo reversible reduction and oxidation, e.g. phenazine-1-carboxamide (Rabaey *et al*., 2005) or pyocyanin which was extracted from *P. aeruginosa* strains and was found to enhance the power output of the MFC (Rabaey *et al*., 2004).

### Current generation source

**Figure 4:**
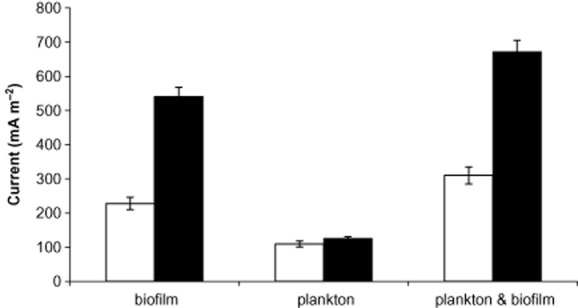
Current density obtained in MFCs with biofilm only, plankton only and plankton with biofilm. MFC operated with acetate (black bar); with phenol (light bar).

The current generated in the MFC may be attributed to the anode biofilm redox activity or to a postulated soluble natural mediator molecule which may be released into the medium by the bacterial cells. At the end of the experiments the anode with the attached biofilm was exchanged by a sterile electrode (without biofilm) which was introduced into the existing plankton bacterial cells (viable count of 2 × 10^9^ cells ml^−1^ in 450 ml) in order to examine each approach. It is important to indicate that during 5 h of the current measurement, the reabsorbed bacterial cells were negligible [viable count of 8 × 10^1^ cells ml^−1^ (3 ml)]. Conversely, the anode covered with the biofilm [viable count of 4 × 10^10^ cells ml^−1^ (30 ml)] was inserted into a sterile medium of another MFC in order to measure the contribution of the biofilm to the current. The current was measured 5 h after the anodes were exchanged, in the MFCs which were operated using either acetate or phenol (Fig. 4).

The current density measured from the plankton bacterial cells with a fresh electrode was 125 and 109 mA m^−2^ for the MFC operated using acetate and the MFC operated using phenol respectively. In contradistinction, the current obtained with biofilm-covered electrodes in sterile medium was 541 and 228 mA m^−2^ under the same resistor of 1000 Ω and identical experimental configuration. These results show that both the plankton bacterial cells and the biofilm contributed to the overall MFC voltage.

### Morphological examination using scanning electron microscope (SEM) and confocal scanning laser microscopy (CSLM) analyses

**Figure 5:**
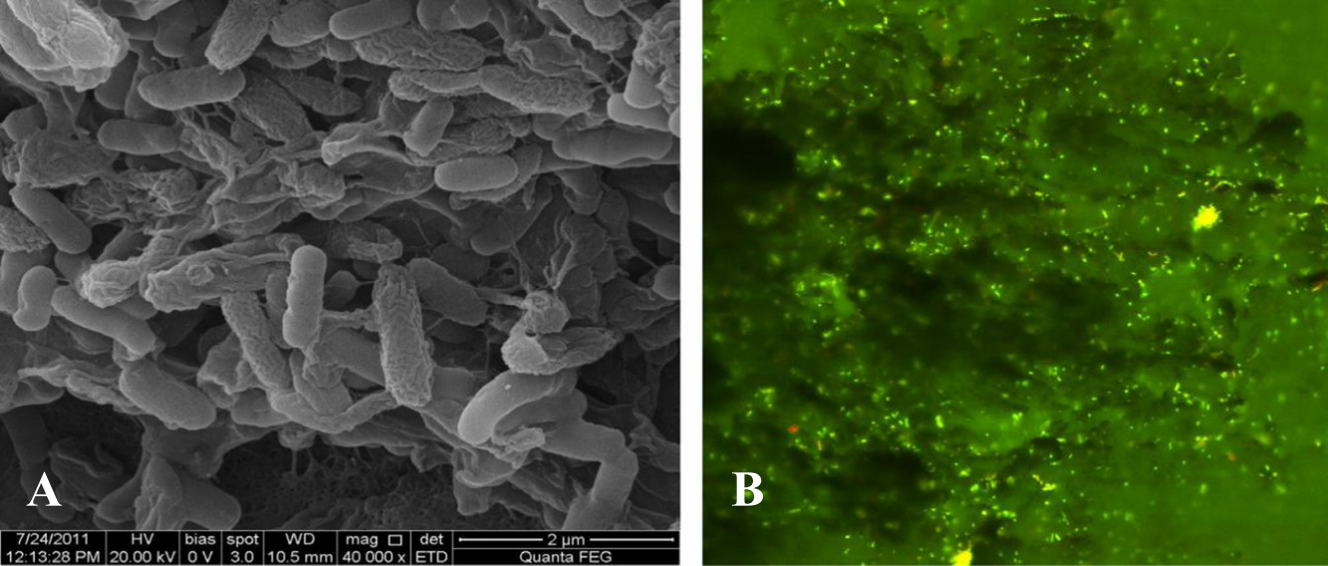
*Cupriavidus basilensis* biofilm grown on a graphite anode in MFC supplied with phenol as a sole carbon source. SEM micrograph, magnification of 40 000 (A) and CSLM micrograph, magnification of 40 (B).

The biofilm attached to the graphite rod working electrode in the MFC was examined by SEM analysis. At the end of the experiment using phenol as the sole carbon source, 1 cm of the graphite electrode was cut and the attached biofilm was fixed with glutaraldehyde. The SEM images show a biofilm of *C. basilensis* on the graphite electrode (Fig. 5A). In addition, the biofilm on the anode was stained with LIVE/DEAD BacLight viability kit, and analysed by CSLM. This analysis revealed that the majority of the biofilm composed of live cells (Fig. 5B).

A biofilm current associated with direct electron transfer has been found in select bacterial strains such as *Rhodoferax ferrireducens* (Chaudhuri and Lovley, 2003) and *G. sulfurreducens* (Bond and Lovley, 2003). A *G. sulfurreducens* strain KN400, which was recovered from a biofilm on the anode in MFC that was operated for 5 months, showed extensive adherence to a graphite anode compared with the wild type (Yi *et al*., 2009). Reguera *et al*. indicated that *G. sulfurreducens* live population was preferentially located in direct contact with the anode surface, and that the dead cells were present primarily in the upper biofilm layers (Reguera *et al*., 2006).

The results showing the biofilm formation on the graphite anode (Fig. 5A and B) support the experiment which showed that the biofilm was responsible for 109 mA m^−2^ when the MFC was operated with phenol as a sole carbon source. Similar results obtained in MFC using acetate as a sole carbon source.

### Phenol degradation in the MFC

**Figure 6:**
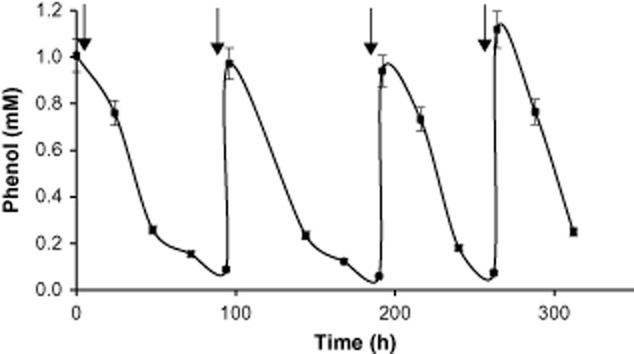
Phenol degradation in the MFC. Phenol concentration was measured during MFC operation. The arrows indicate phenol additions to a final concentration of 1.06 mM.

Phenol degradation by *C. basilensis* in the MFC was examined and compared with controls: MFC(OCP), abiotic MFC (same configuration and operation as the MFC but without bacterial cells) and a MFC kept under anaerobic conditions during the entire experiment. Phenol at a final concentration of 1.06 mM was added to the anode compartment at 0, 96, 192 and 264 h (labelled by the black arrows) and its concentration was measured during the entire experiment (Fig. 6). The results depict almost complete degradation of phenol in the MFC which was operated under a low oxygen concentration. The phenol degradation rate in the first two feedings was 0.95 mg l^−1 ^h^−1^ on average, while in the fourth feeding the phenol degradation rate increased to 1.32 mg l^−1 ^h^−1^. These results are in line with the current produced during the same time intervals shown in Fig. 1. After 200 h, there was a significant increase in current production (Fig. 1), which was correlated with the higher phenol degradation rate (Fig. 6). The phenol degradation rate in the control MFC(OCP) was about the same as in the MFC (data not shown). We assume that the low concentration of oxygen that penetrated through the cap of the bottle and the feeding with fresh medium enabled the *C.* *basilensis* bacterial cell to utilize the phenol which led to bacterial growth. However, there were no bacterial growth current formation and phenol degradation in the MFC which was operated under anaerobic conditions. In the abiotic MFC, phenol (1.06 mM) was added only at the beginning of the experiment. In this MFC, the phenol concentration was 0.97 ± 0.03 mM during the entire experiment. The 8% decrease in phenol concentration is attributed to evaporation.

Luo *et al*. showed phenol (400 mg l^−1^) degradation in an air-cathode MFC which was inoculated with mixed aerobic and anaerobic bacteria collected from a municipal wastewater treatment plant. The degradation rate of phenol in the MFC increased about 15% compared with the MFC(OCP). The degradation efficiency of phenol in the MFC reached above 95% within 60 h (Luo *et al*., 2009).

## Conclusions

Operating a MFC with a pure culture of *C basilensis* using a minimal medium containing acetate or phenol under low dissolved oxygen led to a current generation up to 902 and 310 mA m^−2^ respectively. In both experiments, the majority of the current was obtained from the biofilm bacterial cells. After 72 h in the MFC, phenol removal was 86%, while in the control MFC it was 64%. Furthermore, SEM analysis showed a developed biofilm and CSLM analysis revealed a live population this biofilm.

In this study we demonstrated, for the first time, the use of *C. basilensis* facultative aerobe bacterial cells in a MFC which led to electricity generation and phenol degradation.

## Experimental procedures

### Minimal medium (MM)

One litre of MM composed of: 0.42 g of KH_2_PO_4_, 0.22 g of K_2_HPO_4_, 0.2 g of NH_4_Cl, 0.38 g of KCl, 0.36 g of NaCl, 0.04 g of CaCl_2_·2H_2_O, 0.1 g of MgSO_4_·7H_2_O, 1.8 g of NaHCO_3_, 0.5 g of Na_2_CO_3_, 2.04 g of NaC_2_H_3_O_2_·3H_2_O, 10.0 ml of a vitamin solution and 10.0 ml of trace mineral solution.

### Trace mineral solution

2.14 g of nitriloacetic acid, 0.1 g of MnCl_2_·4H_2_O, 0.3 g of FeSO_4_·7H_2_O, 0.17 g of CoCl_2_·6H_2_O, 0.2 g of ZnSO_4_·7H_2_O, 0.3 g of CuCl_2_·2H_2_O, 0.005 g of H_3_BO_3_, 0.09 g of Na_2_MoO_4_, 0.11 g of NiSO_4_·6H_2_O and 0.2 g of Na_2_WO_4_·2H_2_O in 1 l of deionized water.

### Vitamin solution

Two milligrams of biotin, 2 mg of folic acid, 10 mg of pyridoxine HCl, 5 mg of thiamine HCl, 5 mg of riboflavin, 5 mg of nicotinic acid, 5 mg of d-ca-pantothenate, 0.1 mg of vitamin B12, 5 mg of p-amionobezoic acid and 5 mg of lipoic acid, in 1 l of deionized water.

### MM-phenol (MM-P) and MM-acetate (MM-A)

MM which containing phenol (1.06 mM) or acetate (10 mM) as an electron donor and 1 mM cysteine as a reductant was indicated as MM-P and MM-A respectively. Acetate and phenol concentrations of 10 mM and 1.06 mM respectively were chosen from preliminary experiments that showed that these concentrations are optimal for a long period of bacterial growth. All reagents and chemicals of analytical grade were purchased from Sigma-Aldrich, Israel.

### Bacterial strain and growth conditions

A pure culture of *C. basilensis* bacterial cells (9750) was purchased from DSMZ, Germany. The bacterial cells were grown MM-A or MM-P in a sealed bottle at 26°C with agitation of 100 r.p.m. The MFC was inoculated with a log phase culture of *C. basilensis* to a final absorbance of 0.2 OD_600_ in 450 ml of MM-A or MM-P in the anode chamber (volume of 500 ml). The MFC with the bacterial cells was operated at 26°C, the anode compartment was agitated slowly using a magnetic stir bar. The bacterial absorbance in the anode compartment was measured at 660 nm using a spectrophotometer (GENESYS 10S UV-VIS, Thermo Scientific, USA) at 600 nm.

### MFC set-up

The MFC was comprised of a dual-glass chamber separated by a proton-selective membrane (Nafion® 115; Ionpower, USA). The volume of each chamber was 500 ml. The anode chamber had four ports on the top screw cup for solution sampling, feeding, a graphite rod working electrode (3 mm diameter and 3.2 cm length) (Graphite Engineering and Sales, Greenville, MI) and a reference electrode Ag/AgCl (CHInstruments, USA). The cathode top had two ports for the counter electrode (2 cm × 2 cm carbon cloth ELAT-LT-1400 W) (ETEK International, USA) and for aeration. The cathode electrode was brush coated with a catalyst composite layer of 0.5 mg Pt m^−2^ (Johnson Matthey, USA) from a slurry containing a weight ratio of 8:1:1 Pt : Nafion (5% wt solution, Ionpower, USA) : carbon (Vulcan XC72 Cabot, USA). The two electrodes were connected by a copper wire lead and the junction was protected from corrosion by imbedding using commercial silicon. All parts were autoclaved prior to each experiment, except for the reference electrode (Ag/AgCl electrode in 3 M KCl from CHInstruments) which was rinsed with 70% ethanol followed by sterile water. The MFC chamber was filled with 450 ml of sterile medium containing 350 ml of MM and 100 ml of phosphate buffer, pH 6.9 in partial aerobic conditions (the oxygen was not excluded from the medium however, the anode chamber with a volume of 500 ml was filled with 450 ml medium in a sealed bottle). The final carbon source at the beginning of the experiment was 10 mM acetate or 1.06 mM phenol. Every 100 h, 100 ml from the anode chamber was replaced by fresh medium (MM) and the acetate or phenol was added to a final concentration of 10 mM or 1.06 mM respectively. The MFC was placed in a thermostatic bath at 26°C and the anode chamber was agitated slowly using a magnetic stir bar. The anode and the cathode were connected through an external resistor 1000 Ω (Resistance Decade Box 72-7270, Tenma, USA). The cathode chamber was aerated through a 0.45-μm-pore-size filter (Whatman, USA) to maintain an oxygenated environment while preventing contamination. A parallel identical control MFC (held at OCP) was constructed in each experiment which was maintained under the same conditions but was not connected to a resistor. Two control abiotic MFCs were constructed, one was connected to a resistance of 1000 Ω to measure residual currents and the second was held at OCP and served to analyse phenol electro-oxidation under MFC conditions. In addition, an anaerobic MFC was designed similarly to the MFC, except of that the medium was flushed with N_2_-CO_2_ (80:20) to remove oxygen before autoclaving in sealed bottles and during the entire experiment this MFC maintained an anaerobic environment by continuously supplying N_2_.

### Dissolved oxygen measurement

The dissolved oxygen was measured at the beginning, before supplying fresh medium (every 100 h) and at the end of the experiment, using CyberScan DO 110 Dissolved Oxygen meter, EUTECH instruments, Singapore.

### Steady-state current – voltage polarization

External resistances were exerted on the MFC, stepping from 100 000 to 1000 Ω. The steady voltages (versus the reference electrode) and currents of the anode, the cathode and the complete cell were measured at each resistance step after 10 min.

### CV measurements

CV measurements were performed at the beginning, at the end of each experiment and after centrifuged the medium at 15 000 *g* for 10 min, using a 6K10 Sigma centrifuge, (USA). A CHI760 potentiostat, (CHInstruments, USA) was used to study the oxidation and reduction reactions on the anode surface at a potential scan rate of 10 mV s^−1^ (minimum of five scans) using the original electrodes of the MFC.

### Phenol concentration measurement

The phenol concentration was determined by a colorimetric method using 4-aminoantipyrine similarly to the method described in Greenberg (2005). The phenol concentration was measured as follows: Solution A: 0.05 N NH_4_OH in H_2_O, Solution B: 0.1 M phosphate buffer, pH 6.8. Solution C: 2% of 4-aminoantipyrine in H_2_O. Solution D: 8% of K_3_Fe(CN)_6_ in H_2_O. An examined sample of 10 μl was stirred with 990 μl H_2_O, followed by addition of 25 μl of solution A, adjusted to pH 7.9, followed by addition of solution B (2 μl), 10 μl of solution C and 10 μl of solution D. The sample was mixed and left at room temperature for 15 min. The OD of the samples was measured at 500 nm using a spectrophotometer (GENESYS 10S UV-VIS, Thermo Scientific, USA). The same procedure was performed for blank controls in which 10 μl of H_2_O was added instead of the examined sample. A phenol concentration calibration curve was constructed using phenol solutions with pre-defined concentrations (0–10 mM in H_2_O).

### Phenol degradation in MFC

Phenol (1.06 mM) was added to the poised MFC as well as to the control MFCs at indicated times and its concentration was measured periodically. The abiotic MFC was inoculated with phenol only at the beginning of the experiment. The abiotic control was used to measure evaporation of phenol from the anode and phenol electrooxidation.

### Viable count of plankton bacterial cells and biofilm adsorbed to the anode

The anode covered with biofilm bacterial cells was washed with PBS and placed in a tube containing PBS. Removal of the bacterial cells was performed by bath sonication (Transsonic 460/H). The PBS with the detached bacterial cells was diluted and the appropriate dilutions were pour plated on NB agar plates followed by incubation at 37°C for 24 h. Viable cells were determined by counting the colony-forming units (cfu) and multiplying them by the appropriate dilution. Viable counts of the plankton bacterial cells were performed by serial dilution in PBS and pour plated on NB agar as described above.

### Examine the biofilm on the anode electrode using two methods

#### Scanning electron microscope (SEM)

The biofilm cells attached to 1 cm (length) of a graphite anode from the MFC and MFC(OCP) were washed and fixed with 2% glutaraldehyde for 2 h, followed by 1% osmium tetroxide. The cells were then dehydrated by incubation in increasing concentrations of ethanol. The specimens were gold-coated using a SPUTTER COATER, LKB 8800A Instrument, Australia. Scanning was performed with a JEOL 840 scanning electron microscope at an accelerating voltage of 20 kV.

#### Confocal scanning laser microscopy (CSLM)

In order to examine the viability of a biofilm on the anode by CSLM, the anode was removed from the fuel cells at the end of the experiment, washed with phosphate-buffered saline, attached to the microscope slide with scotch tape, fluorescently stained using a Live/Dead Kit L7007 (Molecular Probes, Israel) for microscopy and quantitative assays, and examined with a Zeiss LSM 700.

### Statistics

Each experiment was performed at least in triplicate. All primary data are presented as means ± standard deviations of the mean.
